# Mechanistic insights into Nipah virus 5′ UTR functionality reveal an antiviral target

**DOI:** 10.1099/jgv.0.002141

**Published:** 2025-08-29

**Authors:** Lishi Liu, Chaohu Pan, Zhen Chen, Fang Zhang, Wuxiang Guan, Aiping Zeng, Haojie Hao

**Affiliations:** 1 Center for Emerging Infectious Diseases, Wuhan Institute of Virology, Center for Biosafety Mega-Science, Chinese Academy of Sciences, Wuhan, Hubei, 430071, PR China; 2 Hubei Jiangxia Laboratory, Wuhan, Hubei, 430200, PR China; 3 Key Laboratory of Plant Germplasm Enhancement and Specialty Agriculture, Wuhan Botanical Garden, Chinese Academy of Sciences, Wuhan, Hubei, 430074, PR China; 4 National Clinical Research Center for Infectious Diseases, Shenzhen Third People’s Hospital, Southern University of Science and Technology, Shenzhen, PR China; 5 Department of Ophthalmology, Union Hospital, Tongji Medical College, Huazhong University of Science and Technology, Wuhan, PR China

**Keywords:** 5′ UTRs, alternative translation, antiviral intervention, leaky scanning, Nipah virus (NiV)

## Abstract

The highly pathogenic Nipah virus (NiV), a World Health Organization priority pathogen with pandemic potential, remains a critical public health threat due to its capacity to cause fatal encephalitis and respiratory disease. Despite its 1998 emergence, no approved therapeutics exist against NiV infection, underscoring the urgent need to identify genomic regulatory elements as antiviral targets. Our study focuses on the extended 5′ UTRs characteristic of NiV transcripts, a distinctive genomic feature whose functional significance remained enigmatic. Comparative reporter assays showed these UTRs strongly inhibit downstream ORF translation through mechanisms distinct from internal ribosome entry site activity. Mutagenesis studies identified upstream ATG elements in multiple transcripts as critical regulators of translational efficiency, with the C 5′ UTR exhibiting maximal suppression. A functional hotspot spanning within the C 5′ UTR was mapped as the primary ribosomal initiation blockade, and ribosome leaky scanning was confirmed as the mechanism enabling dual-protein expression. Notably, therapeutic targeting of this regulatory element with antisense oligonucleotides significantly impaired viral replication. These findings provide fundamental insights into henipaviral translation regulation while identifying concrete antiviral targets, particularly the druggable C 5′ UTR element, advancing efforts to combat this biosafety level 4 pathogen.

## Data Availability

All the data generated during the current study are included in the manuscript.

## Introduction

Nipah virus (NiV) is a bat-borne zoonotic henipavirus that causes severe and often lethal respiratory and neurological disease in humans [1]. NiV, an enveloped single-stranded RNA virus, belongs to the genus *Henipavirus* of the family *Paramyxoviridae* [1,2] and was first identified during an outbreak of severe febrile encephalitis in Malaysia and Singapore [3,4]. Subsequent outbreaks have occurred in Bangladesh, northeastern India and the Philippines [1,5,10]. NiV infection can spread between humans, causing severe respiratory symptoms and fatal neurological symptoms [11]. Case fatality rates for these outbreaks ranged from 40 to 92%, with many survivors experiencing long-term neurological sequelae [3,6]. In addition to its high lethality, NiV uses ephrin-B2 and -B3 receptor tyrosine kinases for host cell entry, allowing a broader host range compared to other paramyxoviruses [12,13]. In 2018, NiV was listed as one of the key diseases that could cause serious international outbreaks by the World Health Organization (WHO) Development Blueprint. Despite ongoing research efforts, no effective vaccines or therapies for NiV disease have been licensed, highlighting the urgent need for effective countermeasures against NiV infection [2].

Like other viruses in the family *Paramyxoviridae*, the NiV genome follows the ‘integral multiple of 6 principle’, which means that the genome can only replicate and transcribe efficiently when the number of bases is a multiple of six [14]. The negative-sense, non-segmented RNA genome of NiV is 18,246 nt in length and contains 6 transcription units that encode 6 structural proteins, nucleocapsid (N), phosphoprotein (P), matrix protein (M), fusion protein (F), glycoprotein (G) and polymerase (L), and three non-structural proteins: V, W and C [15]. The N, P and L proteins are required for both transcription and replication of the genome [14]. The V and W proteins are produced by RNA editing of the *P* gene mRNA from the NiV polymerase protein and share the same N-terminal amino acids as the P protein [16,17]. The C protein, which can antagonize the interferon response and regulate viral replication, is expressed from an alternative ORF embedded in the *P* gene and shares no sequence similarity with the P protein [18,21]. In the *Paramyxovirinae*, the alternative translation mechanisms of C proteins include leak scanning [17,22, 23], ribosomal shunting [24], non-AUG initiation [25,26] and proteolytic processing [27]. However, the mechanism of NiV C protein expression remains unclear.

Viruses rely on the host cell’s translational machinery to synthesize their proteins. According to canonical eukaryotic translation initiation, a given mRNA can only synthesize a single protein [28]. However, multiple proteins of the viruses should be expressed to complete the life cycle with the limited genome. To overcome this challenge, viruses have evolved a number of strategies, such as producing monocistronic subgenomic RNAs [29,30] and encoding long polyproteins that can be cleaved into multiple proteins by host or viral proteases [31,32]. These strategies allow viruses to produce multiple proteins but have limitations. If proteins are produced from monocistronic subgenomic RNAs, the virus must ensure that all subgenomes are packaged into a single viral particle, which is challenging and often results in non-infectious particles. If multiple proteins are produced from a long polyprotein, the relative amounts of proteins cannot be regulated efficiently, as structural proteins often need to be produced in larger quantities than enzymes. Thus, viruses have evolved a variety of strategies including internal ribosome entry site (IRES), leaky scanning, non-AUG initiation, ribosome shunting and reinitiation [33]. These non-classical modes of translation initiation help viruses overcome classical translation challenges and allow for regulation at the translational level. Leaky scanning involves alternative AUG codons in the 5′ UTR that interfere with the translation of the primary ORF (pORF) [34,35]. The efficiency of initiation at the upstream start codon directly relies on its context; if the context of the first AUG on the message is suboptimal, more ribosomes will continue scanning until they encounter a downstream start codon [36,37].

NiV poses a severe public health threat due to its high mortality rate and potential for outbreaks, yet no Food and Drug Administration (FDA)- or WHO (World Health Organization) -approved antiviral therapies exist to combat infections. Current research has prioritized investigational agents targeting key stages of the viral lifecycle, including monoclonal antibodies (mAbs) and small-molecule inhibitors [38]. Neutralizing mAbs 1F5 and 12B2 have shown protective efficacy in animal models and have been utilized on a compassionate-use basis, yet comprehensive clinical trials remain to be completed [39]. Small-molecule compounds targeting viral fusion proteins (G and F) or the RNA-dependent RNA polymerase (L), such as remdesivir [40,41] and favipiravir [42], have exhibited moderate anti-NiV activity *in vitro* and animal studies, though their therapeutic efficacy remains limited. Antisense oligonucleotides (ASOs) have emerged as a promising therapeutic approach through their ability to specifically target viral RNA via Watson–Crick base pairing, triggering degradation or blocking translation [43]. This platform offers advantages such as rapid design, high specificity and adaptability to viral mutations [44]. However, no published studies to date have investigated ASO-based interventions against NiV infection.

This study reveals key translational control mechanisms in NiV 5′ UTRs. The C, G and L 5′ UTRs strongly suppress downstream translation via upstream ATG (uATG) elements, with the C 5′ UTR showing maximal inhibition. Ribosomal leaky scanning drives alternative P/C protein synthesis. Targeting the C 5′ UTR’s regulatory region with ASOs effectively blocked viral replication. These findings not only advance our understanding of paramyxoviral translation regulation but also provide a framework for developing precision antivirals against high-containment pathogens through RNA-targeted intervention strategies.

## Methods

### Cell line

HEK293T [CRL-11268, American Type Culture Collection (ATCC)] cells and Vero (CCL-81, ATCC) were cultured in Dulbecco’s Modified Eagle’s Medium (C11995500BT, Gibco) supplemented with 10% FBS (Gibco) at 37 °C with 5% CO2.

### Viruses

NiV strains (NiV-MY) were obtained from the Microorganisms and Viruses Culture Collection Center at the Wuhan Institute of Virology, Chinese Academy of Sciences. Viruses were amplified in Vero cells, and the indicated TCID_50_ were titrated in Vero cells, following the Reed–Muench method. NiV infection was conducted with Multiplicity Of Infection (MOI) = 0.01 for Vero cells.

### Plasmid construction

Plasmid pBi-IRES2 was the plasmid pmCherry-IRES2-EGFP originally reported by Liu *et al*. (shown as Fig. 5b) [45], which was constructed by inserting the mCherry gene with an N-terminal 3×Flag tag into the IRES-active vector pIRES2-EGFP (Clontech). The plasmids pBi-NiV-N-5′ UTR, pBi-NiV-P-5′ UTR, pBi-NiV-C-5′ UTR, pBi-NiV-M-5′ UTR, pBi-NiV-F-5′ UTR, pBi-NiV-G-5′ UTR and pBi-NiV-L-5′ UTR were constructed by inserting the 5′ UTR sequence of the respective NiV transcripts (synthesized based on National Center for Biotechnology Information (NCBI) Reference Sequence: NC_002728.1) into the pmCherry-EGFP vector between the mCherry and Enhanced Green Fluorescent Protein (EGFP) coding sequences. For the plasmids shown in Fig. S1 (available in the online Supplementary Material), an additional 100 bp of the corresponding coding sequence was inserted downstream of each 5′ UTR.

Construction of EGFP reporter plasmids. pCDNA3Del plasmid was constructed by deleting 68 nt prior to the KpnI restriction enzyme site on pcDNA3 to shorten the distance between the CMV promoter and the KpnI restriction enzyme site. The parent reporter plasmid pcDNA3Del-EGFP was generated by inserting the EGFP between the KpnI and NotI restriction enzyme sites. The plasmids pNiV-N, pNiV-P, pNiV-C, pNiV-M, pNiV-F, pNiV-G, pNiV-L and pActin were constructed by introducing each of NiV 5′ UTR (synthesized based on NCBI Reference Sequence: NC_002728.1) and *β*-actin 5′ UTR into pcDNA3Del-EGFP between the KpnI restriction enzyme site and EGFP ORF.

5′uORF mutant plasmids. The pNiV-C-muATG was constructed by mutating nt 2,406 to G. The pNiV-F-muATG was constructed by mutating nt 6,489 and nt 6,496 to G and G, pNiV-G-muATG was constructed by mutating nt 8,784 and nt 8,815 to G and T and pNiV-l-muATG was constructed by mutating nt 11,278 and nt 1,1371 to G and T.

Constructs to analyse the effect of nucleotides between C 5′ UTR uATG and primary ATG (pATG) on downstream EGFP expression. The pNiV-C-5' UTR was constructed by inserting the NiV C 5' UTR into an EGFP reporter plasmid . pNiV-C-5′ UTR-amp-1, pNiV-C-5′ UTR-amp-2 and pNiV-C-5′ UTR-amp-3 were constructed by replacing nt 2,409–2,427 with heterologous sequences from the ORF of ampicillin spanning from nt 16–34, nt 382–400 and nt 752–770 based on the plasmid pNiV-C-5'UTR, respectively. pNiV-C-5′ UTR -mut-1 and pNiV-C-5′ UTR -mut-2 (Fig. S2) were generated by replacing nt 2,409–2,427 with scrambled versions of the original sequence using the *NovoPro* online tool.

Constructs for investigating the alternative translation mechanism of the C protein. The plasmid of pCMV14-NiV-P-mRNA was generated by inserting the full-length NiV P mRNA sequence (excluding the termination codon) into the pCMV14 vector. The plasmid of pCMV14-NiV-P was constructed by mutating the natural stop codon (TAG) of the *C* gene to TTG on pCMV14-NiV-P-mRNA, and the downstream sequence 5′- AACTATCTACTACAGGACTGAATCCCACAGCAGTACCGTTCACTCTGAGAAACCTGTCTGATCCTG-3′ was replaced with a 3×Flag tag sequence 5′- GACTACAAAGACCACGACGGAGATTACAAAGATCACGACATCGACTAC

AAGGACGACGACGACAAG-3′. pCMV14-NiV-P-mATGp, pCMV14-NiV-P-kozak and pCMV14-NiV-P-antikozak plasmids were constructed by mutating the *P* gene ATG, 5′-ATTCATCCA-3′ and 5’- ATTCATCCA−3′ to GUG, 5′-GCCGCCACC-3′ and ATATATTTT based on the plasmid pCMV14-NiV-P, respectively.

### Plasmid transfection

Transfection was performed when HEK293T cells reached 60–80 % confluence 18–24 h post-seeding. Lipofectamine 2000 (Invitrogen) reagent and plasmid DNA were separately diluted in Opti-MEM at a 1:1 ratio (µl/µg), incubated for 5 min at room temperature, then combined and mixed by gentle inversion. Following a 5-min incubation to form lipoplexes, the lipoplex solution was added dropwise to the cells, followed by horizontal rocking to ensure even distribution. Cells were maintained at 37 °C with 5% CO2 until harvest at experimentally determined time points.

### Western blot

Cell lysates were collected 48 h post-transfection and were subjected to 12% Tris-glycine SDS-PAGE gels, followed by transferring to nitrocellulose membranes. Protein detection was carried out with anti-GFP (Proteintech, 66002), anti-Flag (Sigma, F1804), anti-NiV-N (Alpha Diagnostic, NIV21-A), anti-GAPDH (Proteintech, 60004-1-lg) and anti-*β*-actin (Santa Cruz Biotechnology, sc-47778) according to the standard protocols. Luminescent signals were detected with the ChemiDoc™ MP 235 imaging system (Bio-Rad).

### ASO transfection

Vero cells were plated in a 12-well plate (~5×10³ cells) the day before, and the next day, transfection was performed using TransIT-X2 reagent (Mirus, China) when the cells reached 80% confluency. First, ASO (synthesized by Beijing Tsingke Biotech Co., Ltd.) was added to Opti-MEM, mixed gently with a pipette tip, followed by the addition of 15 µl TransIT-X2 reagent. After mixing, the solution was briefly centrifuged and allowed to stand for 20 min before adding the transfection mixture to the cells.

### Quantitative reverse transcription PCR

Total RNA was isolated from cultured cells using TRIzol reagent (Invitrogen) following the manufacturer’s protocol. Reverse transcription and quantitative Polymerase Chain Reaction (qPCR) were subsequently performed using the HiScript II One Step qRT-PCR SYBR Green Kit (Vazyme) in Hard-Shell 96-well PCR plates (Bio-Rad Laboratories). Amplification and detection were carried out on a CFX Connect Real-Time PCR Detection System (Bio-Rad Laboratories) with the following cycling conditions: 55 °C for 5 min, 95 °C for 30 s, and 40 cycles of 95 °C for 10 s and 60 °C for 45 s. The primers used in quantitative reverse transcription PCR were as follows: NiV N (forward: 5′-AGGAGATGGAAGGCTTGATG-3′, reverse: TGCTCATGTCTGTTATCCGTAG); EGFP (forward: 5′-TGAGCAAAGACCCCAACGAG-3′, reverse: 5′-CTTGTACAGCTCGTCCATGC-3′); GAPDH (forward: 5′-GAAGGTGAAGGTCGGAGTC-3′, reverse: 5′-GAAGATGGTGATGGGATTTC-3′); NiV-P-300 (forward: 5′-AAATTGGAACTAGTCAATGATGGC-3′, reverse: 5′-ATCGTCCGTATGTCTTCTGTATTT-3′).

### ASO binding assay

300-nt RNA transcripts were generated through T7 *in vitro* transcription (MEGAshortscript™ Kit, AM1333, Thermo Fisher, USA) using PCR-amplified template DNA, which was obtained by amplifying plasmid pCMV14-NiV-P with T7 promoter-linked primers (forward: 5′-TAATACGACTCACTATAGAGGATCCAAGAGATTTACTCTAGG-3′; reverse: 5′-CTTAGACATTCCCCCCTCAACTTGT-3′). The transcribed RNA (5 µg) was denatured at 75 °C for 5 min and immediately chilled on ice for 1 min, then incubated with 0.2 µM ASO and pre-washed NeutrAvidinTM Agarose (Thermo, 29201) in 1×TBS buffer at 4 °C for 2 h with gentle rotation. After incubation, the RNA-ASO-bead complexes were washed eight times with 1×TBS buffer (10×TBS, Beyotime, ST661), and the bound RNA was extracted using TRIzol reagent (Invitrogen) following the manufacturer’s protocol. Finally, the ASO-bound RNA levels were quantified by qPCR using sequence-specific primers.

### Statistical analysis

Statistical analysis of the histogram was performed using an unpaired t-test in GraphPad Prism (version 8.0). Data are presented as means±sd (*n*=3). A *P*-value of 0.05 or less was considered statistically significant.

## Results

### NiV 5′ UTRs do not possess IRES activity

**Fig. 1.**
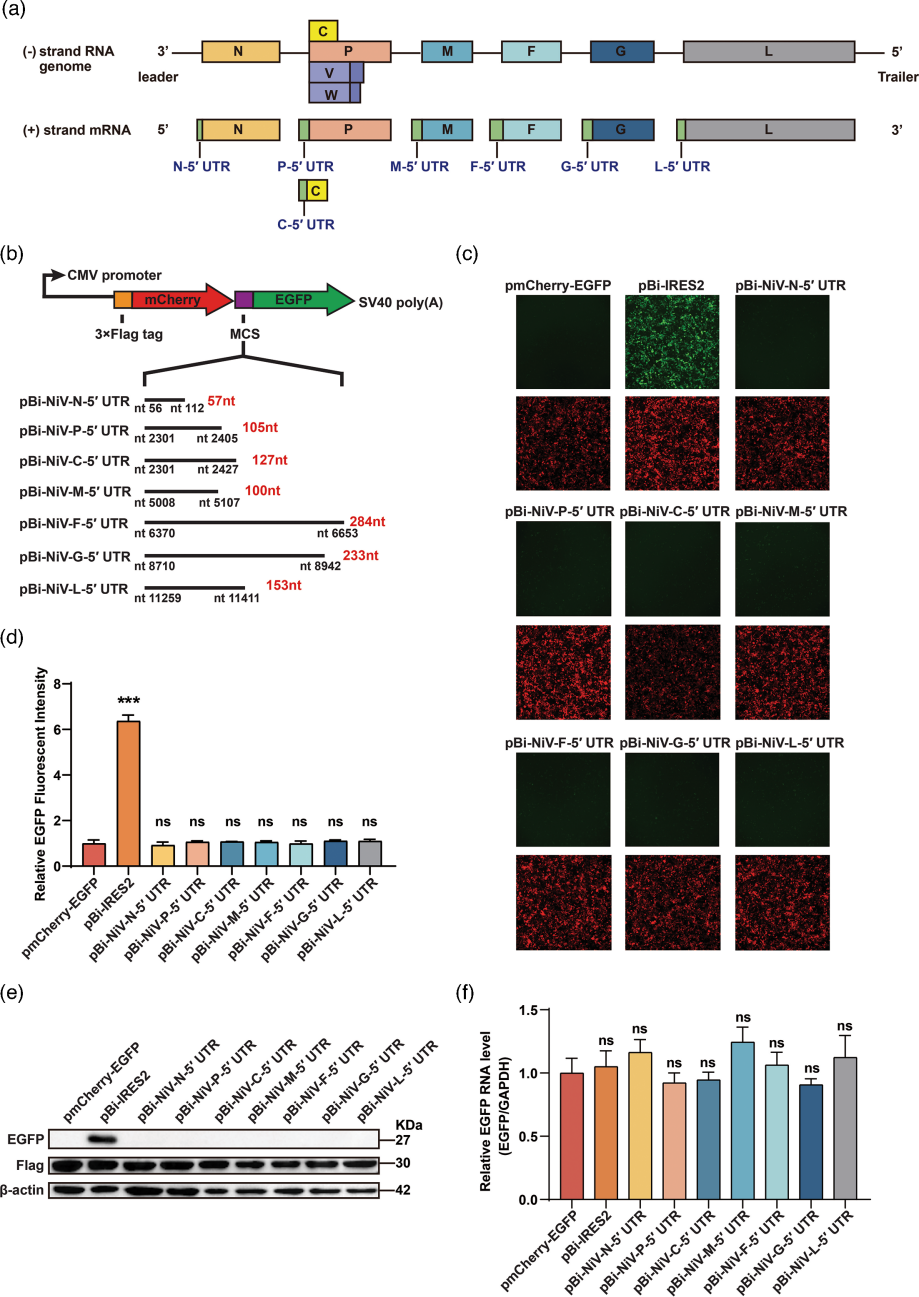
The NiV 5′ UTR does not have IRES activity. (**a**) Schematic representation of the NiV negative-sense RNA genome and the corresponding positive-sense viral mRNAs, with the 5′ UTRs indicated as parts of the respective mRNAs. (**b**) A diagram of the bicistronic reporter vector used. MCS, multiple cloning site; NiV 5′ UTRs were cloned into the MCS. (**c**) Equal amounts of the plasmids described in (a) were transfected into HEK293 T cells. Forty-eight hours post-transfection, the expression of EGFP and mCherry was measured via microscopy. (**d**) Quantification of EGFP fluorescence in (c) using ImageJ. Data are mean ± sd (*n* = 3); ***P < 0.001; ns, not significant; one-way ANOVA with Dunnett’s multiple comparison test. (**e**) EGFP expression corresponding to (c) was analysed by Western blot using an anti-EGFP antibody. Flag was used as a marker for mCherry expression, and *β*-actin served as the loading control. (**f**) EGFP mRNA levels from the (c) treatment groups were measured by qPCR using EGFP-specific primers. Data are mean ± sd (*n* = 3); ns, not significant; one-way ANOVA with Dunnett’s multiple comparison test.

Host cells may inhibit viral replication by suppressing cap-dependent translation. To bypass this, some viruses use IRES elements in the 5′ UTR to initiate translation independently [46]. NiV encodes nine viral proteins from six transcriptional units, requiring tight translational control for efficient replication (Fig. 1a). Its 5′ UTRs, ranging from 57 to 284 nt, can form secondary structures that may influence translation. To evaluate whether NiV 5′ UTRs exhibit IRES-mediated translational activity, we designed a bicistronic reporter plasmid with a multiple cloning site (MCS) between mCherry and EGFP ORF. As depicted in Fig. 1b, each of the NiV 5′ UTRs was inserted into the MCS. These plasmids were transfected into HEK293T cells, and the signals of mCherry and EGFP were measured by microscopy and Western blot at 48 h post-transfection. Compared to pBi-IRES2 (known as pmCherry-IRES2-EGFP), which contains a well-characterized IRES element [45], none of the NiV 5′ UTRs drove the expression of EGFP (Fig. 1c, d), consistent with Western blot results (Fig. 1e). To determine whether the lack of EGFP expression was due to differences in RNA levels, we performed qPCR analysis. The results showed no significant difference in EGFP RNA level downstream of the NiV 5′ UTRs, excluding transcript abundance as a confounding factor (Fig. 1f) and suggesting that the observed differences were due to translational regulation. To further examine whether the downstream coding sequence influences potential IRES activity, we constructed additional reporter plasmids by inserting each NiV 5′ UTR along with the following 100 bp of its coding region (Fig. S1A). These constructs were transfected into HEK293T cells, and mCherry and EGFP signals were assessed using a high-content imaging system. As shown in Figs S1B and S1C, EGFP fluorescence was absent in all constructs, while mCherry expression remained unaffected. This result was again confirmed by Western blot analysis (Fig. S1D). Moreover, qPCR analysis revealed no significant differences in GFP RNA levels (Fig. S1E), further supporting the conclusion that NiV 5′ UTRs lack IRES activity.

### NiV 5′ UTRs regulate downstream EGFP reporter translation

**Fig. 2.**
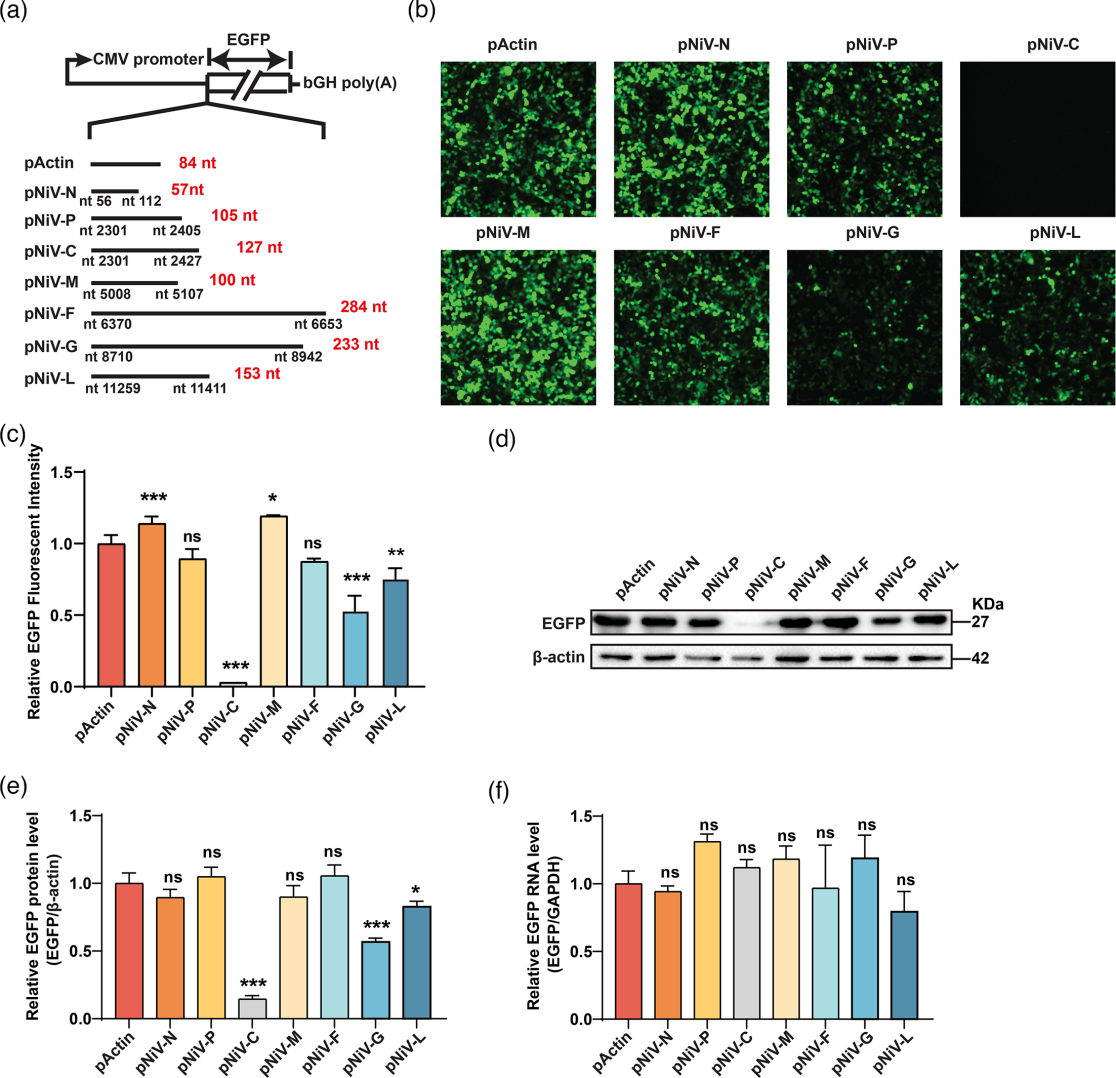
The NiV C, G and L 5′ UTR suppress the expression of downstream EGFP. (**a**) A diagram of the reporter vector and NiV 5′ UTR was placed upstream of the EGFP. (**b**) Equal amounts of the plasmids described in (a) were transfected into 293 T cells. The expression of EGFP was measured at 48 h post-transfection via a fluorescence microscope. (**c**) Quantification of EGFP fluorescence in (b) using ImageJ. Data are mean ± sd (*n* = 3); *P < 0.05, **P < 0.01, ***P < 0.001; ns, not significant; one-way ANOVA with Dunnett’s multiple comparison test. (**d**) The protein level of EGFP was measured using anti-EGFP antibody, with *β*-actin serving as the loading control. (**e**) Grey-scale analysis of EGFP/*β*-actin Western blot bands from (d), quantified using ImageJ. Data are shown as mean ± sd (*n* = 3). Statistical significance was assessed using one-way ANOVA with Dunnett’s multiple comparison test; *P < 0.05, ***P < 0.001; ns, not significant. (**f**) EGFP mRNA levels corresponding to the conditions shown in (c) were measured by qPCR. Data are mean ± sd (*n* = 3); ns, not significant; one-way ANOVA with Dunnett’s multiple comparison test.

To assess the impact of NiV 5′ UTRs on the translation of downstream ORFs, plasmids were constructed with each NiV 5′ UTR inserted upstream of the EGFP ORF (Fig. 2a) and transfected into HEK293T cells. EGFP expression was detected at 48 h post-transfection. Immunofluorescence showed that 5′ UTRs from C, G and L reduced EGFP signals compared to the p-*β*-actin control, while the N and M 5′ UTRs enhanced expression (Fig. 2b, c). Consistent with the fluorescence signals, the C, G and L 5′ UTRs decreased EGFP translation, while the other 5′ UTRs did not affect the EGFP translation, as confirmed by Western blot (Fig. 2d, e). To exclude transcript-level differences as a confounding factor, we quantified GFP mRNA by qPCR and observed no significant variation among constructs (Fig. 2f). Together, these data demonstrate that NiV 5′ UTRs modulate the expression of downstream EGFP primarily at the translational level.

### Upstream ORFs in the C, F and L 5′ UTR dramatically suppressed the expression of pORF

**Fig. 3.**
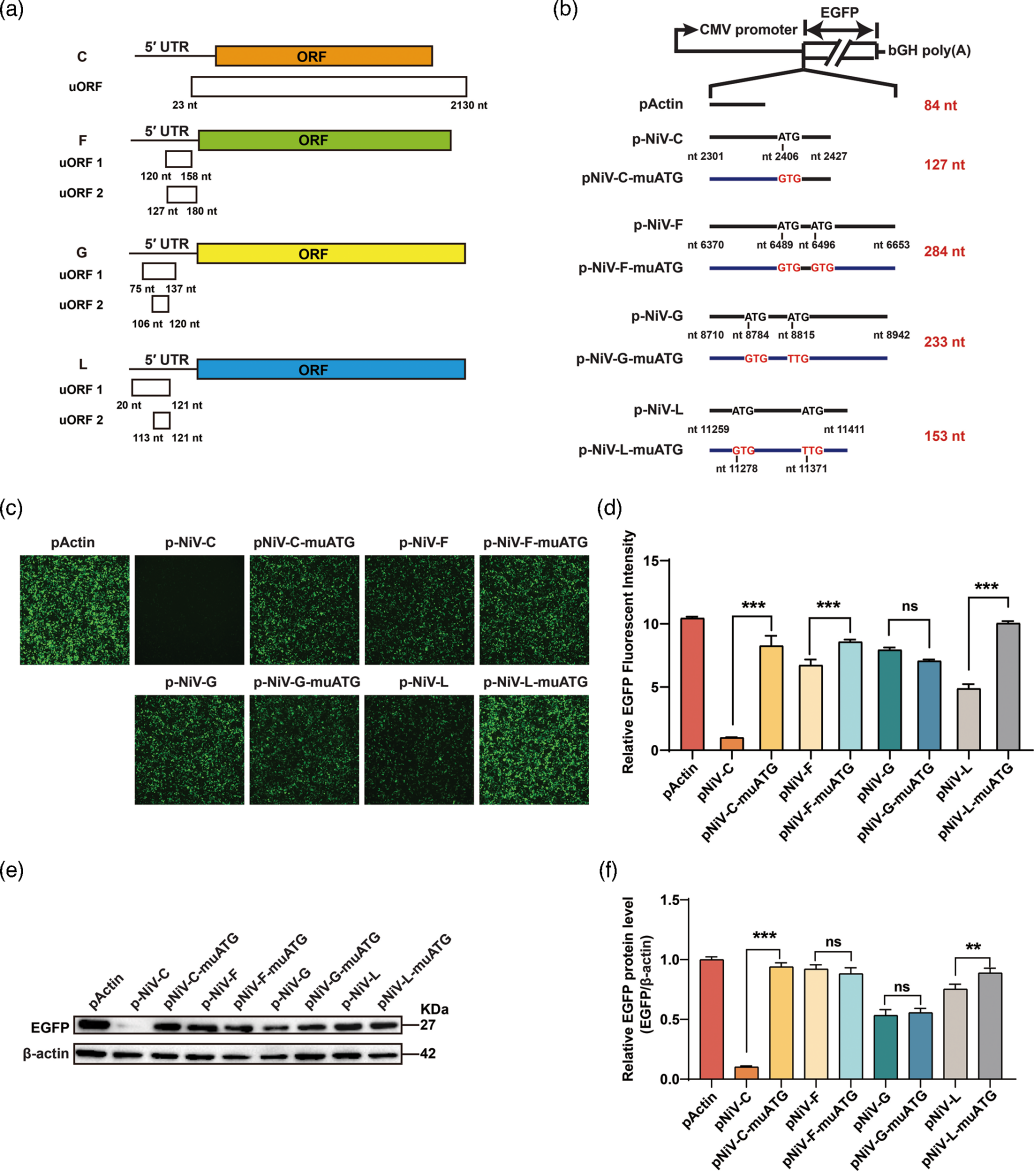
The uORF in the NiV C, F and 5′ UTR modulates the expression of downstream EGFP. (**a**) Schematic structure of uORF and ORF in the mRNAs of NiV C, F, G and L. (**b**) A diagram of the reporter vector and the uATGs in the C, F, G and L was mutated. (**c**) Equal amounts of the plasmids described in (a) were transfected into HEK293T cells, and EGFP was detected by fluorescence microscopy 48 h post-transfection. (**d**) EGFP fluorescence in (c) was quantified using ImageJ. Data are mean ± SD (*n* = 3); ***P < 0.001, ns: not significant; one-way ANOVA with Dunnett’s multiple comparison test. (**e**) EGFP expression in (e) was assessed by Western blot using an anti-EGFP antibody, with *β*-actin serving as the loading control. (**f**) Grey-scale analysis of EGFP and *β*-actin bands from (e), quantified using ImageJ. Data are mean ± sd (*n* = 3); **P < 0.01, ***P < 0.001; ns, not significant; one-way ANOVA with Dunnett’s multiple comparison test.

To investigate how the NiV 5′ UTRs affect the translation of downstream ORFs, we analysed their sequences and found that, unlike P, N and M, NiV C, F, G and L 5′ UTRs contain upstream ORFs (uORFs) (Fig. 3a). Notably, the C 5′ UTR uORF uniquely overlapped with pORF. In the context of C translation, the region between the P and C start codons remains untranslated; however, it is translated when expressed in the context of P translation. As uORFs are known to suppress translation of downstream ORFs [47], their presence was considered a potential cause of the observed translational inhibition. To test this, the uATGs in the C, F, G and L were mutated (Fig. 3b), and the resulting plasmids were transfected into HEK293T cells. At 48 h post-transfection, enhanced EGFP fluorescent signals were detected in constructs with mutated C, F and L 5′ UTRs (Fig. 3c, d). The uATG mutation in the NiV C 5′ UTR led to a significant increase in GFP expression shown in the western results and the analysis (Fig. 3e, f). These data indicate that the uORFs present in the C, F and L 5′ UTRs inhibit translation of the pORF, with the C 5′ UTR showing the most pronounced effect, warranting further investigation.

### Cis-elements in C 5′ UTRs modulate downstream ORF expression

**Fig. 4.**
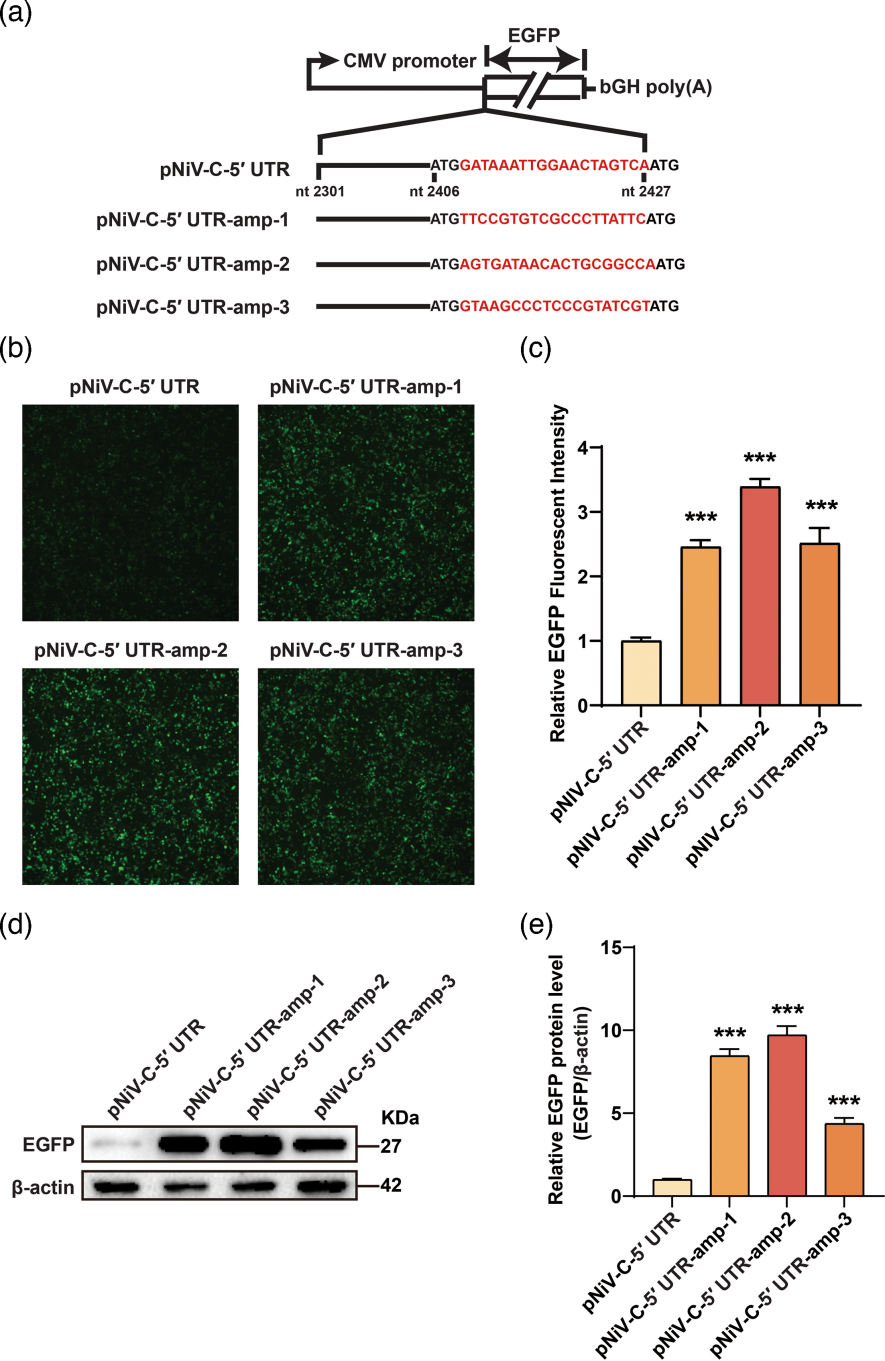
The sequences between the uATG and the pATG inhibited the expression of downstream EGFP. (**a**) A diagram of the reporter vector and the sequences between the uATG and the pATG was replaced with heterologous sequences from the ORF of ampicillin. (**b**) HEK293T cells were transfected with the plasmids in (a), and EGFP fluorescence was imaged by fluorescence microscopy 48 h post-transfection. (**c**) EGFP fluorescence in (b) was quantified using ImageJ. Data are mean ± sd (*n* = 3); ***P < 0.001; one-way ANOVA with Dunnett’s multiple comparison test. (**d**) The expression of EGFP from (b) was measured by Western blot using anti-EGFP antibody. (**e**) Grey-scale analysis of EGFP and *β*-actin bands from (d), quantified using ImageJ. Data are mean ± sd (*n* = 3); ***P < 0.001; ns, not significant; one-way ANOVA with Dunnett’s multiple comparison test.

To explore the regulatory mechanism of NiV C 5′ UTR on downstream ORF translation, three plasmids – pNiV-C-5′ UTR-amp-1, pNiV-C-5′ UTR-amp-2 and pNiV-C-5′ UTR-amp-3 – were generated based on the pNiV-C-5′ UTR. The region between uATG and pATG in the NiV C 5′ UTR was replaced with heterologous sequences derived from the ampicillin resistance gene (Fig. 4a), which naturally relies on a prokaryotic Shine–Dalgarno sequence and lacks eukaryotic translational control elements (e.g. Kozak motifs or IRES). Transfection of these constructs into HEK293T cells resulted in increased EGFP expression compared to pNiV-C-5′ UTR, as shown by fluorescence imaging (Fig. 4b, c) and confirmed by Western blot analysis (Fig. 4d, e). To further validate the regulatory role of this region, two additional constructs – pNiV-C-5′ UTR-mut-1 and pNiV-C-5′ UTR-mut-2 – were generated by randomly scrambling the native sequence between the uATG and pATG (Fig. S2A). When transfected into HEK293T cells, both mutants showed markedly enhanced EGFP expression, as evidenced by fluorescence imaging and Western blotting (Fig. S2E). These data demonstrate that *cis*-elements located between the uATG and pATG in the NiV C 5′ UTR repress translation of the downstream ORF.

### Alternative translation of P and C proteins via ribosomal leaky scanning

**Fig. 5.**
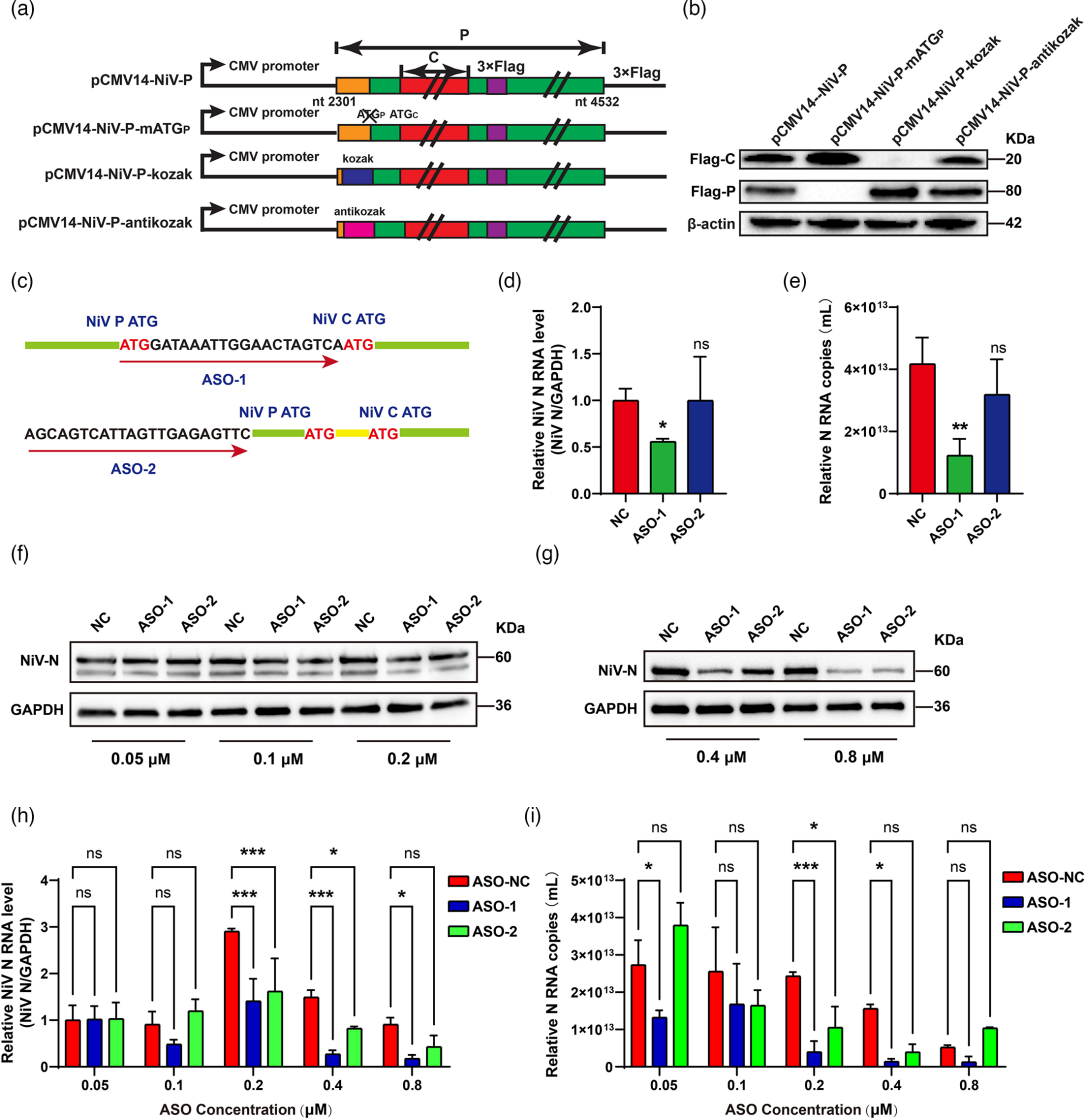
Alternative translation of the P and C proteins is dependent on the mechanism of ribosome leaky scanning. (**a**) A diagram of the pCMV14-NiV-P and its mutants. The start codon of the P protein was mutated in the pCMV14-NiV-P-mATGp. In pCMV14-NiV-P-kozak and pCMV14-NiV-P-antikozak, the Kozak sequences of 5′-ATTCATCCA-3′ were mutated to 5′-GCCGCCACC-3′ and ATATATTTT based on the plasmid pCMV14-NiV-P, respectively. (**b**) Equal amounts of the plasmids described in (a) were transfected into HEK293 T cells. At 48 h post-transfection, the Western blot was performed to detect the expression of protein C and P by using anti-Flag antibody. (**c**) Schematic illustrations of two ASO designs. ASO-1 was designed as a single-stranded DNA targeting the sequence between the NiV P uATG and the C pATG. ASO-2 was designed as a single-stranded DNA complementary to the upstream sequence of the NiV P uATG. (d and e) Vero cells were transfected with ASOs at a final concentration of 0.15 µM for 24 hours post-infection (hpi), followed by NiV infection. Cellular RNA and supernatant were collected at 48 hpi. Viral RNA levels in infected cells (**d**) and viral RNA copy numbers in the supernatant (**e**) were quantified by qPCR. Data are mean ± sd (*n* = 3); *P < 0.05, **P < 0.01; ns, not significant; one-way ANOVA with Dunnett’s multiple comparison test. (**f–i**) Vero cells were transfected with gradient concentrations of ASO-1 and ASO-2 for 24 hpi prior to NiV infection. Proteins, cellular RNA and supernatant were harvested at 48 hpi. Western blot was performed to detect the N protein expression (**f and g**), while intracellular viral RNA levels (**h**) and supernatant viral RNA copy numbers (**i**) were determined by qPCR. Data are mean ± sd (*n* = 3); *P < 0.05, ***P < 0.001; ns, not significant; two-way ANOVA with Dunnett’s multiple comparison test.

The uATG in the C 5′ UTR serves as the start codon for the P protein, with both proteins translated from a single mRNA. The mechanism of multiple proteins expressed from a single mRNA involves ribosomal leaky scanning. According to this model, disrupting the uATG is expected to enhance translation of the downstream ORF. The ATG of the P protein was mutated in the plasmid pCMV14-NiV-P-mATG_P_ (Figs 5a and S3A). Transfection of this construct led to increased C protein expression (Fig. 5b, lane 2). Furthermore, the efficiency of leaky scanning depends on the surrounding sequence context of the uATG. A strong context favours ribosome initiation at the uATG, thereby reducing downstream expression, while a weak context increases ribosomal readthrough. To examine this, Kozak (strong) and anti-Kozak (weak) sequences were introduced around the P protein start codon (Fig. 5a). In comparison to the WT construct (pCMV14-NiV-P), the Kozak-enhanced construct showed increased P protein levels and decreased C protein expression. Surprisingly, the anti-Kozak construct also moderately enhanced P expression and reduced C expression (Fig. 5b), suggesting that the native P start codon context is even weaker than the introduced anti-Kozak sequence. These results confirm that the alternative translation of P and C proteins is regulated by ribosomal leaky scanning.

### Targeting the leaky scanning region with antisense oligonucleotides

Given the regulatory role of the sequence between the uORF and pORF in leaky scanning, this region was evaluated as a potential antiviral target. Two ASOs were designed: ASO-1 targets the region between the P and C start codons, while ASO-2 targets an upstream segment (Figs 5c and S3B). Transfection with ASO-1 significantly reduced NiV protein expression, intracellular viral RNA levels and viral RNA copy numbers in the supernatant (Fig. 5d, e). In contrast, ASO-2 had no significant effect on viral replication. To further validate the antiviral effect of ASO-1, a dose-response experiment was conducted. Increasing ASO-1 concentrations produced a dose-dependent decrease in N protein levels (Figs 5f, g, S3C, D) and in both intracellular and extracellular viral RNA (Fig. 5h–i). In contrast, ASO-2 had little effect at low to mid doses and only inhibited N protein expression at 0.4 and 0.8 µM. At the highest dose (0.8 µM), both ASOs achieved equivalent knockdown, whereas at 0.1–0.2 µM, ASO-1 was markedly more potent than ASO-2. To investigate the differences in inhibition of NiV replication by ASO-1 and ASO-2, a 300-bp RNA fragment encompassing the NiV P and C 5′ UTRs plus the adjacent coding sequence was synthesized by T7 in vitro transcription (Fig. S3E). Binding affinities of biotin-labelled ASO-1 and ASO-2 were then compared by streptavidin-bead capture and qPCR, revealing no significant difference in RNA association (Fig. S3f). In parallel, both ASOs reduced P protein expression in a cellular transfection assay, with ASO-1 demonstrating greater potency (Fig. S3g). Collectively, these data highlight the critical role of this intergenic 5′ UTR region in modulating ribosomal leaky scanning during NiV translation and point to its potential as a novel antiviral target.

## Discussion

**Fig. 6.**
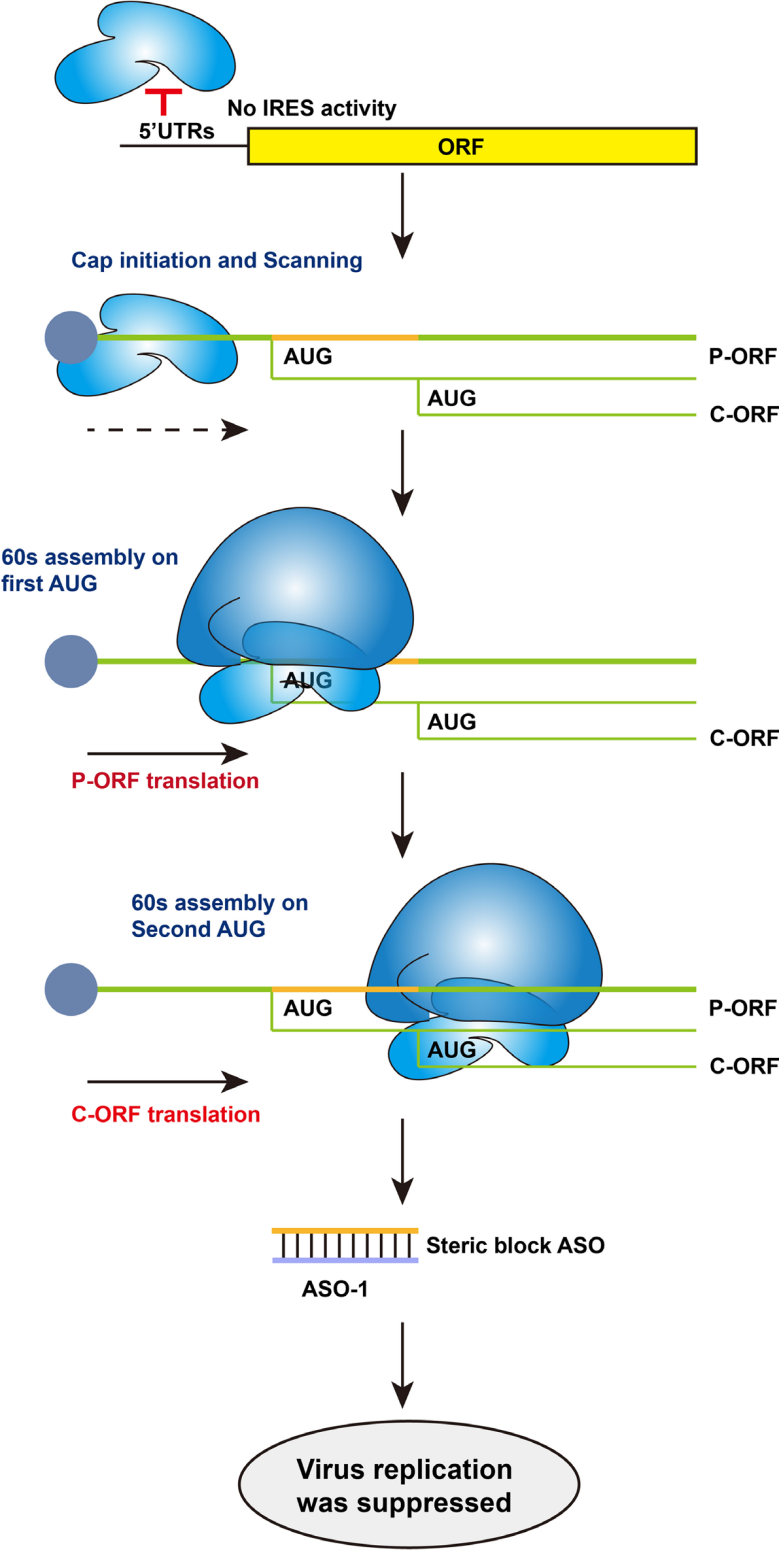
Schematic representation of leaky scanning-mediated translation initiation of Nipah virus P and C proteins. The 5′ UTRs of NiV lack IRES activity, and viral protein translation relies on the 40S ribosomal subunit binding to the 5′ cap for scanning initiation. When the scanning complex encounters the AUG start codon of the P ORF, it recruits the 60S subunit to assemble a functional ribosome and initiates P protein translation. If the flanking sequence context is suboptimal for translation initiation, the 40S subunit continues scanning until it reaches the AUG of the C ORF, where it associates with the 60S subunit to synthesize the C protein. When the designed ASO-1 binds to the regulatory region between the upstream and downstream ORFs, NiV replication is suppressed.

Non-canonical translation mechanisms are critical for many viruses to complete their lifecycle [33]. In this study, we investigated the potential for IRES activity in the 5′ UTRs of NiV. Our findings indicate that NiV 5′ UTRs do not exhibit IRES activity. Using an EGFP reporter system, we discovered that, compared with the *β*-actin 5′ UTR, the 5′ UTRs of the C, G and L genes confer lower translation efficiency of the downstream EGFP. Notably, mutating the uORFs in the C, F, G and L 5′ UTRs revealed that the C 5′ UTR had the most significant impact on EGFP expression. Further analysis identified that the sequences between the uATG and pATG in the C 5′ UTR act as *cis*-elements inhibiting downstream ORF expression. Additionally, we demonstrated that the C and P proteins are translated through a leaky scanning mechanism and that targeting the translational control elements of NiV P/C proteins disrupts viral replication (Fig. 6). This study reveals that NiV utilizes 5′ UTR uORFs and ribosomal leaky scanning to regulate protein synthesis and demonstrates that ASOs targeting this mechanism effectively suppress replication. These findings advance paramyxovirus translational control mechanisms and provide novel antiviral strategies against NiV.

The NiV C protein plays a crucial role in suppressing viral genome replication and antagonizing the interferon response. Recombinant NiV lacking C protein expression loses its virulence [48]. Interestingly, the expression level of the C protein is lower than that of the P, V and W proteins [17], indicating that its production must be tightly regulated. Our experiments confirm that the NiV C protein is regulated at the translational level. The combination of leaky scanning and the *cis*-elements within the 5′ UTR ensures the C protein is expressed at appropriate levels. Future infection experiments will be necessary to validate the leaky scanning mechanism of alternative translation and to understand how the 5′ UTR of the C protein influences NiV replication, interferon antagonism and virulence.

In contrast to the 5′ UTR of *β*-actin, the 5′ UTR of the *M* gene enhances the translation of the EGFP, an effect attributed to the recruitment of eukaryotic elongation factor 1-beta to the M 5′ UTR [49]. Typically, the presence of a uORF in the 5′ UTR inhibits downstream ORF expression [50,51]. Four NiV 5′ UTRs contain uORFs. However, mutating the uORFs in the F, G and L 5′ UTRs had only a modest impact on downstream ORF expression compared to the C 5′ UTR. Further studies are needed to investigate how the 5′ UTRs of the *F*, *G* and *L* genes suppress downstream ORF expression. To further explore this difference, we analysed the Kozak sequences flanking the uORF and main ORF start codons of various NiV genes (Fig. S4). The Kozak sequence is a key determinant of translation initiation efficiency, and stronger Kozak contexts are generally more likely to recruit ribosomes. Interestingly, the uATG of the *NiV-C* gene, which directs P protein translation, is embedded in a strong Kozak consensus sequence (GCCGCCACCATGG) [52], potentially favouring efficient initiation at this site. In contrast, the uATGs of the F, G and L genes are associated with weaker or non-canonical Kozak sequences, which may result in reduced ribosome recognition and leaky scanning past the uORF. This difference may partially explain why the uORF mutation in the *C* gene had a more pronounced effect on downstream protein expression than similar mutations in the *F*, *G* and *L* genes. These observations suggest that both the position and Kozak strength of uORFs are critical factors in determining their regulatory impact on viral gene expression.

Currently, no approved antiviral drugs specifically target NiV infection, and treatment remains largely supportive. Broad-spectrum antivirals such as ribavirin and favipiravir have shown limited efficacy in clinical and experimental settings, often accompanied by significant toxicity or unclear therapeutic windows. mAbs targeting the NiV glycoprotein, while promising, face challenges related to high production costs, potential viral escape mutations and limited accessibility in resource-poor regions where NiV outbreaks typically occur [38]. Additionally, small-molecule inhibitors targeting viral entry or replication often struggle with specificity issues, risking off-target effects on host cellular processes. Our study demonstrates that ASOs can effectively suppress NiV replication. ASOs exhibit distinct advantages over existing antiviral strategies. Their high specificity enables precise targeting of conserved viral RNA sequences, such as the uORF-mediated translational regulatory elements identified in this study, minimizing off-target effects. ASOs can be rapidly designed and synthesized against emerging strains, providing adaptability against viral evolution. Furthermore, chemical modifications such as phosphorothioate backbones and 2′-O-methylation enhance ASO stability and bioavailability [53], overcoming earlier limitations of nucleic acid therapeutics. Their potential for local (e.g. intranasal) or systemic delivery, coupled with lower manufacturing costs compared to biologics like mAbs, makes ASOs a scalable and practical option for NiV outbreaks in endemic areas. By directly disrupting viral translation – a mechanism distinct from current strategies – ASOs may also reduce the likelihood of resistance development, offering a promising addition to the NiV therapeutic arsenal.

The translational regulatory region between uORF and pORF emerged as a promising antiviral target. An ASO designed to disrupt this region (ASO-1) significantly reduced viral protein expression, intracellular RNA levels and viral load in the supernatant, with marked effects even at low concentrations. In contrast, ASO-2, which targets a non-regulatory upstream region, exhibited only modest inhibitory effects, which became apparent at higher concentrations. This suggests that ASO likely exerts its anti-NiV activity by interfering with viral translation rather than RNA degradation or other indirect effects.

These findings have broader implications for antiviral strategies. First, they highlight the importance of uORF-mediated regulation in viral gene expression, a mechanism that could be exploited in other viruses with similar features. Second, the success of ASOs targeting regions directly involved in translation initiation or leaky scanning underscores the potential of RNA-level translational control as a critical antiviral strategy against NiV and related viruses. Future studies should explore the applicability of this strategy in animal models and investigate whether combinatorial targeting of multiple uORF-pORF junctions could enhance antiviral efficacy.

## Conclusions

In summary, this study unravels the critical role of NiV 5′ UTRs in translational regulation and identifies a new antiviral target. By disrupting the uORF-pORF regulatory axis, ASO-based interventions may pave the way for innovative therapeutics against NiV and related viruses.

## Supplementary material

10.1099/jgv.0.002141 Uncited Supplementary Material 1.

## Abbreviations

ASOs: antisense oligonucleotides
ATCC: American Type Culture Collection
EGFP: enhanced green fluorescent protein
F: fusion protein
G: glycoprotein
hpi: hours post-infection
IRES: internal ribosome entry site
L: polymerase
M: matrix protein
mAbs: monoclonal antibodies
MCS: multiple cloning site
N: nucleocapsid
NCBI: National Center for Biotechnology Information
NiV: Nipah virus
P: phosphoprotein
pATG: primary ATG
pORF: primary ORF
qPCR: quantitative Polymerase Chain Reaction
uATG: upstream ATG
uORFs: upstream ORFs
WHO: World Health Organization
