# Supplementary material for: Mechanistic insights into Nipah virus 5′ UTR functionality reveal an antiviral target
Source: J Gen Virol. 2025 Aug 29;106(8):002141. doi: 10.1099/jgv.0.002141 (PMC12396924; doi:10.1099/jgv.0.002141)
Supplement: Uncited Supplementary Material 1. [file jgv-106-02141-s001.pdf]

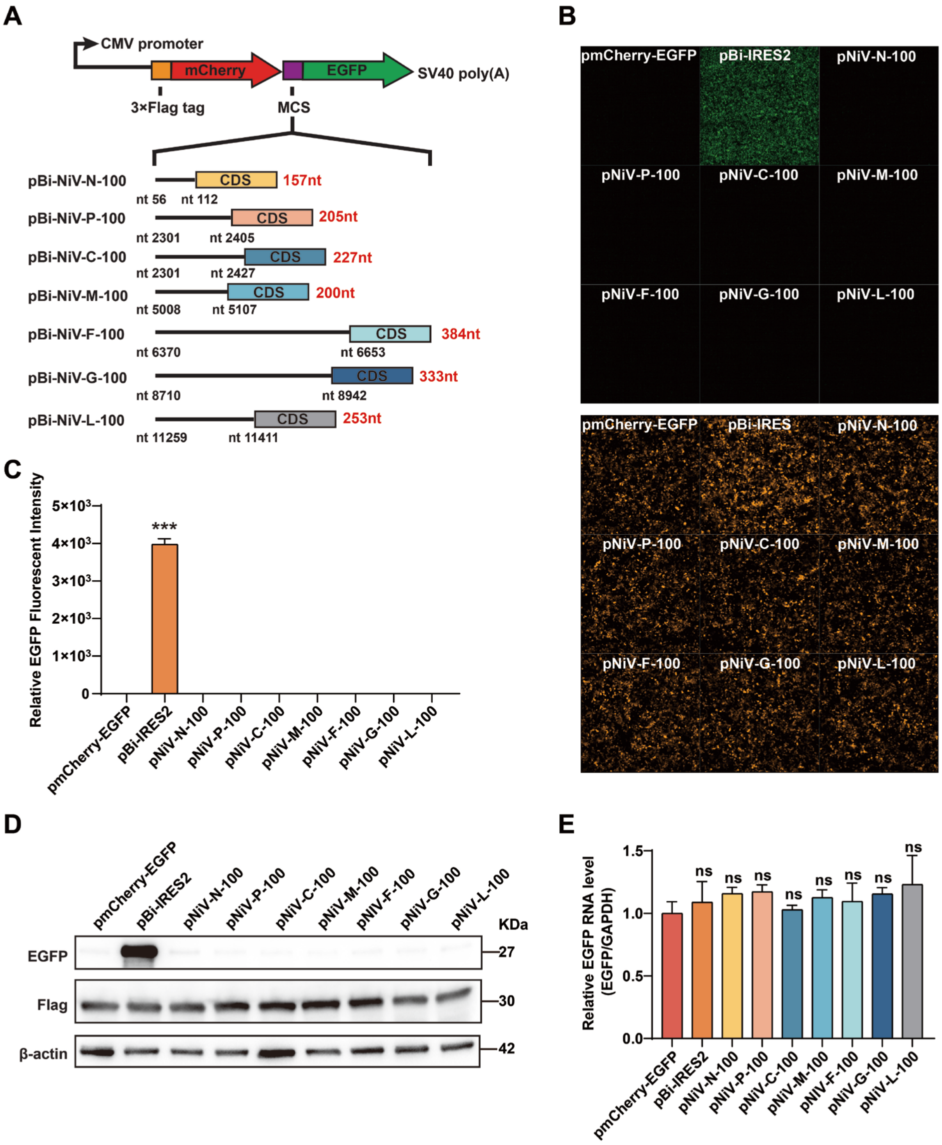

Figure S1. NiV 5′ UTRs do not possess IRES activity independent of CDS sequence.

(A) Diagram of the bicistronic reporter vector used. MCS, multiple cloning site; NiV 5′ UTRs and followed 100bp CDS sequence were cloned into the the MCS. **(B)** Equal amounts of the plasmids described in S1A were transfected into HEK293T cells. 48 h post transfection, the signal of EGFP and Cherry were measured via microscopy. **(C)** Fluorescence quantification of EGFP corresponding to the S1B. Data are mean ± SD (n = 3); ***$p < 0.001$, ns: not significant; one-way ANOVA with Dunnett’s multiple comparisons test. **(D)** The expression of EGFP from S1B was detected by western blotting using anti-EGFP antibody. **(E)** RNA extraction from the HEK293T cells transfected with equal amounts of plasmids was performed for qPCR analysis using EGFP specific primers. Data are mean ± SD (n = 3), ns: not significant; one-way ANOVA with Dunnett’s multiple comparisons test.

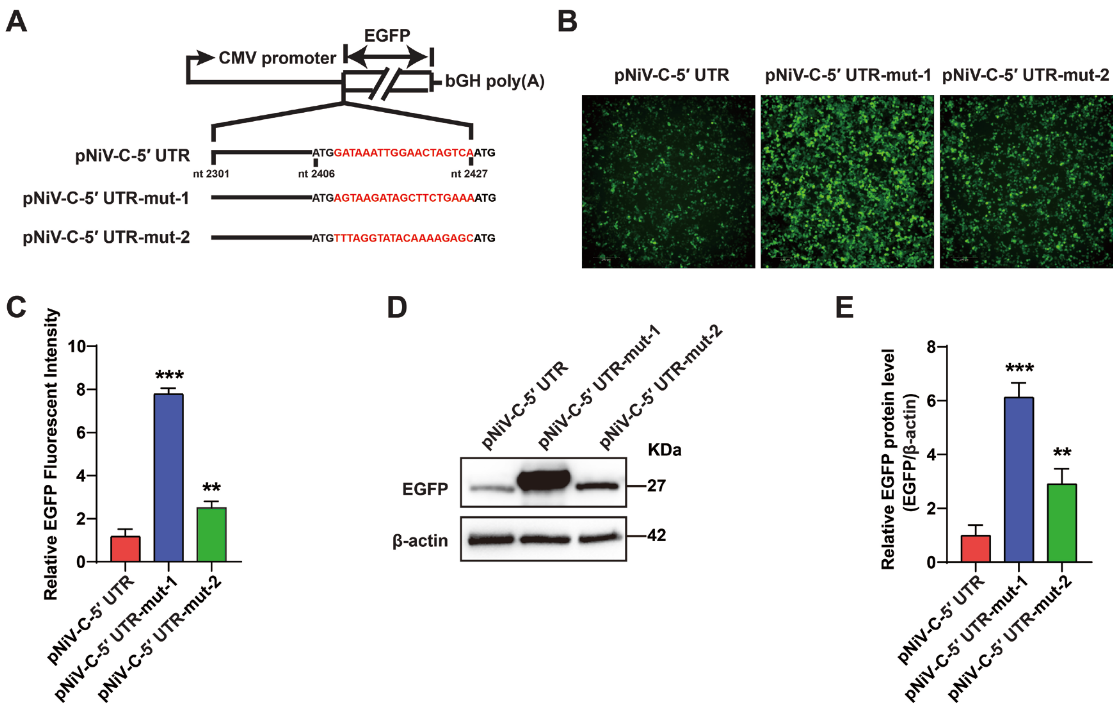

Figure S2. The sequences between the uATG and the pATG of NiV C inhibited the expression of downstream EGFP.

(A) Diagram of the reporter vector and the sequences between the uATG and the pATG were replaced with randomized upset of the original sequence. **(B)** Equal amounts of the plasmids described in S2A were transfected into HEK293T cells. The signal of EGFP was detected by fluorescence microscope at 48 h post transfection. **(C)** Fluorescence quantification of EGFP corresponding to the S2B. Data are mean ± SD (n = 3); **p < 0.01, ***p < 0.001, one-way ANOVA with Dunnett's multiple comparisons test. **(D)** The expression of EGFP from 4B was measured by western blot using anti-EGFP antibody. **(E)** Gray-scale analysis of EGFP and β-actin bands from Figure S2D, quantified using ImageJ. Data are mean ± SD (n = 3); **p < 0.01, ***p < 0.001; one way ANOVA with Dunnett's multiple comparisons test.

A

Plasmid	Sequence
pCMV14-NiV-P	AATTCATCCAATGGATAAATTGG
pCMV14-NiV-P-mATGP	AATTCATCCAGTGGATAAATTGG
pCMV14-NiV-P-kozak	AGCCGCCACCATGGATAAATTGG
pCMV14-NiV-P-antikozak	AATATATTTTATGGATAAATTGG

B

Name	Sequence
NC	CCGAACGTGTCA
ASO-1	AGTTCCAATTTA
ASO-2	CTCAACTAAT

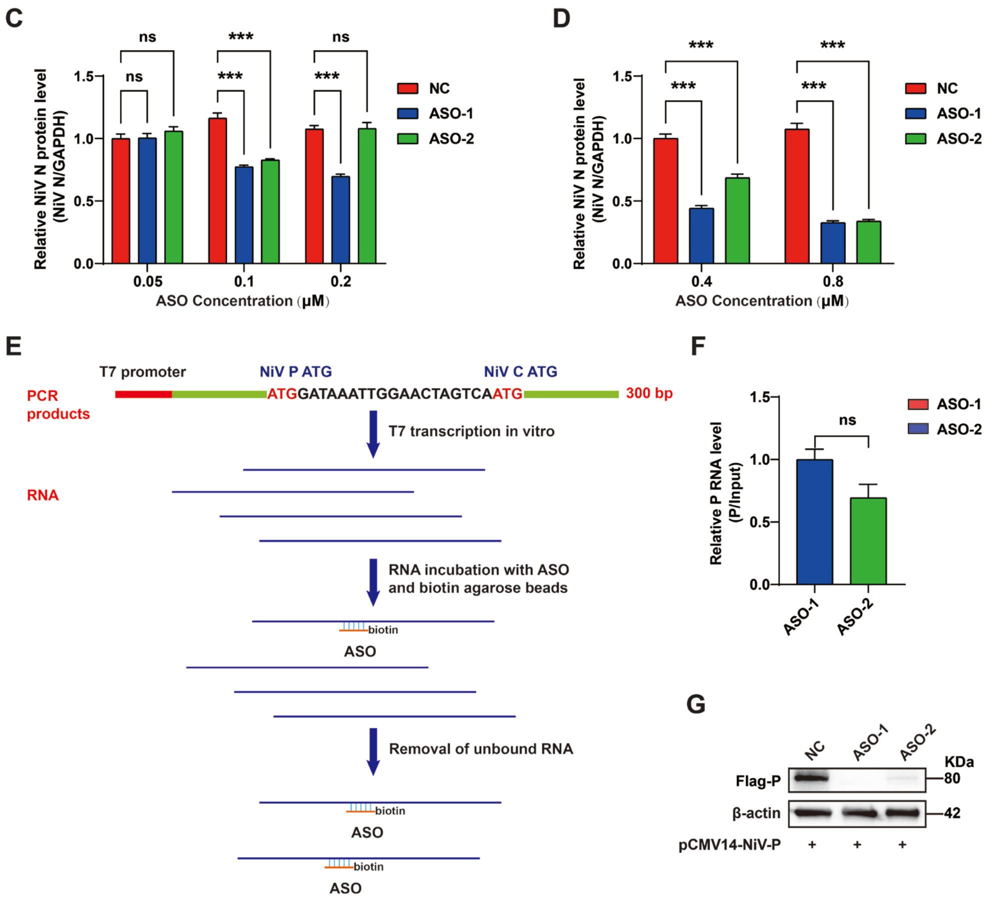

Figure S3. ASOs targeting the 5′ UTR s of NiV P and NiV C inhibit viral replication.

(A) Tabular representation of the sequences spanning positions –10 to +10 relative to the P start codon in pCMV14-NiV-P and its mutants. **(B)** Tabulated sequences of NC, ASO-1, and ASO-2. Each antisense oligonucleotide (ASO) contains five 2'-O-methoxyethyl (2'-MOE) modified bases at both the 5′ and 3′ ends, and the entire backbone is phosphorothioate-modified. **(C&D)** Gray-scale analysis of western bands from Figure 5F and 5G obtained by Image J quantification. Data are mean ± SD (n = 3); ***$p < 0.001$, ns: not significant; two-way ANOVA with Dunnett's multiple comparisons test. **(E)** Schematic diagram of the experimental procedure to assess ASO binding to NiV P RNA. 300-bp PCR products containing the P 5'UTR and C5'UTR sequences were amplified using specific primers incorporating the T7 promoter. The resulting RNA, generated by in vitro transcription with T7 polymerase, was incubated with biotinylated beads along with 0.2 µM ASO-1 and ASO-2. After removing unbound RNA through washing, the amount of bead-bound RNA was quantified using qPCR with specific primers. **(F)** Levels of target RNA bound by ASO-1 and ASO-2 were detected by qPCR according to the experimental procedure in S3E. Data are mean ± SD (n = 3); ns: not significant; unpaired student's t test. **(G)** 0.2 µM ASO and 1 µg of plasmid pCMV14-NiV-P per well were transfected into 12-well plate HEK293T cells. The expression of NiV-P was measured by western blotting using anti-Flag antibody at 48 h post transfection.

NiV gene	start codon	kozak sequence
NiV-C	uATG	GCCGCCACCATGG
	ATG	AACTAGTCAATGA
NiV-F	uATG-1	CCTGGTTTTATGT
	uATG-2	TTATGTTGAATGA
	ATG	GACTCGACAATGG
NiV-G	uATG-1	AGAGATTGAATGC
	uATG-2	CATTACTATATGT
	ATG	TTCAAGAAAATGC
NiV-L	uATG-1	TCCTTGATTATGC
	uATG-2	TTAATTTATATGA
	ATG	AAAACAAAAATGG

Figure S4. Tabular representation of the Kozak sequences flanking the uORF and main ORF start codons of various NiV genes.
